# An overlooked cause of forefoot pain in athletes: Avascular necrosis of the lateral hallucial sesamoid

**DOI:** 10.1016/j.radcr.2025.05.105

**Published:** 2025-06-21

**Authors:** Jochen Gerstner Saucedo, Raghav Pai, Juan Bernardo Gerstner

**Affiliations:** a University of Colorado, Anschutz Medical Campus, Aurora, Colorado, USA; b Department of Radiology, University of Miami, Miami, Florida, USA; c Department of Orthopedics and Traumatology, Clinica Imbanaco, Cali , Valle del Cauca, Colombia

**Keywords:** Avascular necrosis, Hallucial sesamoid, Sesamoid bone, MRI, Forefoot pain, Metatarsalgia

## Abstract

Avascular necrosis (AVN) of the hallucial sesamoids is a rare and often overlooked source of forefoot pain, particularly in athletic populations. We present a case of a 39-year-old female athlete with a 1-year history of progressive pain localized to the plantar aspect of the left first metatarsophalangeal (MTP) joint. The pain had intensified over the previous 6 weeks, especially during weight-bearing activities. Notably, the patient had undergone surgical excision of the right lateral sesamoid 20 years earlier due to a similar diagnosis, which is visible on current postoperative radiographs. Radiographic imaging showed sclerosis and fragmentation of the left lateral sesamoid. MRI confirmed AVN. The patient underwent sesamoidectomy combined with a biplanar Chevron osteotomy to correct moderate hallux valgus and offload the preserved medial sesamoid. Histopathological analysis confirmed the diagnosis. The postoperative recovery was successful, with complete resolution of symptoms. This case emphasizes the necessity of including avascular necrosis in the differential diagnosis of chronic forefoot pain. The case also demonstrates the importance of combining targeted excision and realignment osteotomy in individuals with recurrent or bilateral disease to improve biomechanics and preserve joint function.

## Introduction

Avascular necrosis (AVN) of the hallucial sesamoids is a rare but significant cause of persistent forefoot pain, especially in active individuals and athletes. These small, rounded bones, which are embedded within the flexor hallucis brevis tendon, play a crucial biomechanical role in weight transfer and joint stabilization. AVN can affect either of the sesamoids; however, the lateral sesamoid is much less commonly affected [1,2].

Diagnosis is often delayed due to the extensive differential diagnosis for metatarsalgia [3,4]. While histological confirmation is definitive, other imaging methods, such as CT scans or magnetic resonance imaging (MRI), are essential for early identification, in addition to plain radiographs [2,5,6]. Treatment may include conservative approaches, but surgical intervention may be necessary in cases that do not respond to these methods.

## Case presentation

A 39-year-old female long-distance runner presented with a 1-year history of progressively worsening pain in the plantar region of the left first metatarsophalangeal (MTP) joint. Over the previous 6 weeks, the pain had increased in severity, especially when jogging during the push-off phase. She pursued conservative treatment approaches, such as rest, nonsteroidal anti-inflammatory drugs, and orthotic support, yet did not achieve substantial symptom relief.

The patient had a prior history of AVN of the right lateral hallucial sesamoid, which was surgically removed around 20 years ago, resulting in no complications or residual pain. The current postoperative radiographs distinctly demonstrate the surgical site and the lack of the right lateral sesamoid.

Physical examination revealed localized tenderness over the plantar surface of the left great toe in the area of the lateral sesamoid. Mild soft tissue swelling and a moderate hallux valgus deformity were noted. The first MTP joint retained a full range of motion, but dorsiflexion reproduced pain. There was no varus or valgus hindfoot deformity, and no retraction of the Achilles tendon or gastrocnemius muscles, excluding equinus as a contributing factor.

Plain radiographs of the left foot, including AP, oblique, and lateral views, demonstrated sclerosis, fragmentation, and irregularity of the lateral hallucial sesamoid, suggestive of AVN [6].

**Fig. 1:**
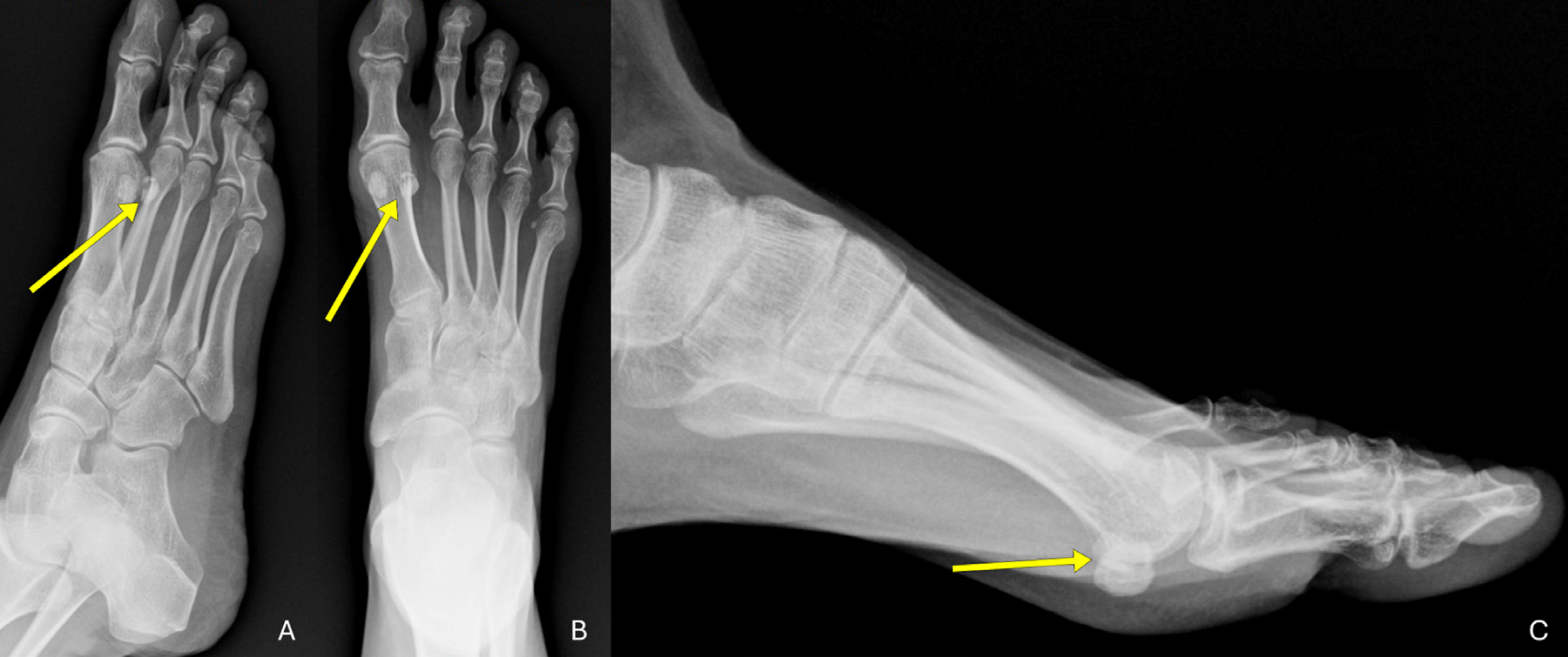
(A) Anteroposterior (AP), (B) oblique, and (C) lateral radiographs of the left foot. Yellow arrows indicate sclerosis and fragmentation of the lateral hallucial sesamoid, suggestive of AVN.

**Fig. 2:**
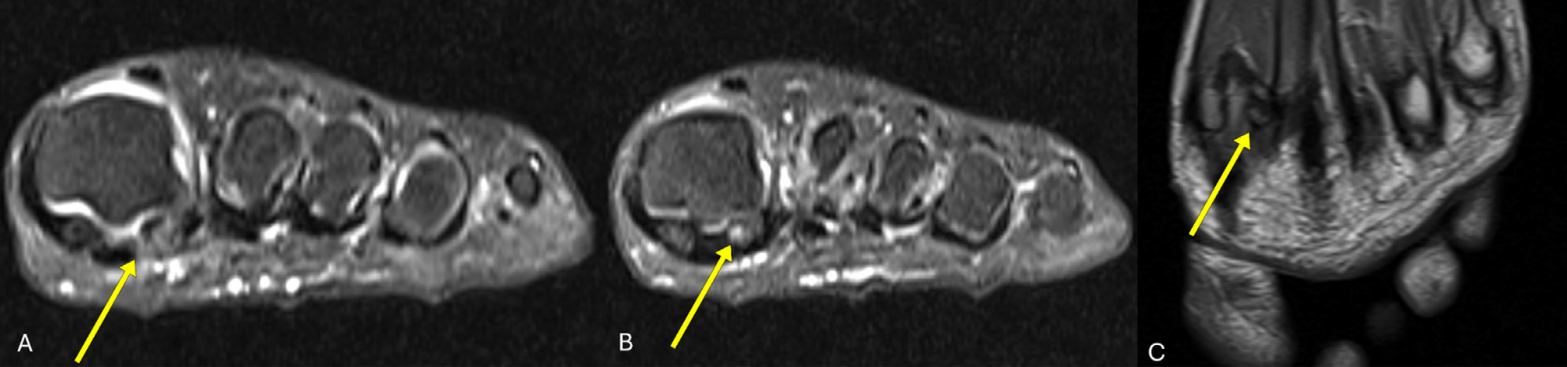
(A) and (B) Axial fat-suppressed proton density (PD-FS) images and (C) coronal T1-weighted image of the left forefoot. Yellow arrows indicate increased signal intensity of the lateral hallucial sesamoid on PD-FS, consistent with bone marrow edema and compatible with AVN. The T1-weighted image demonstrates corresponding hypointensity.

**Fig. 3:**
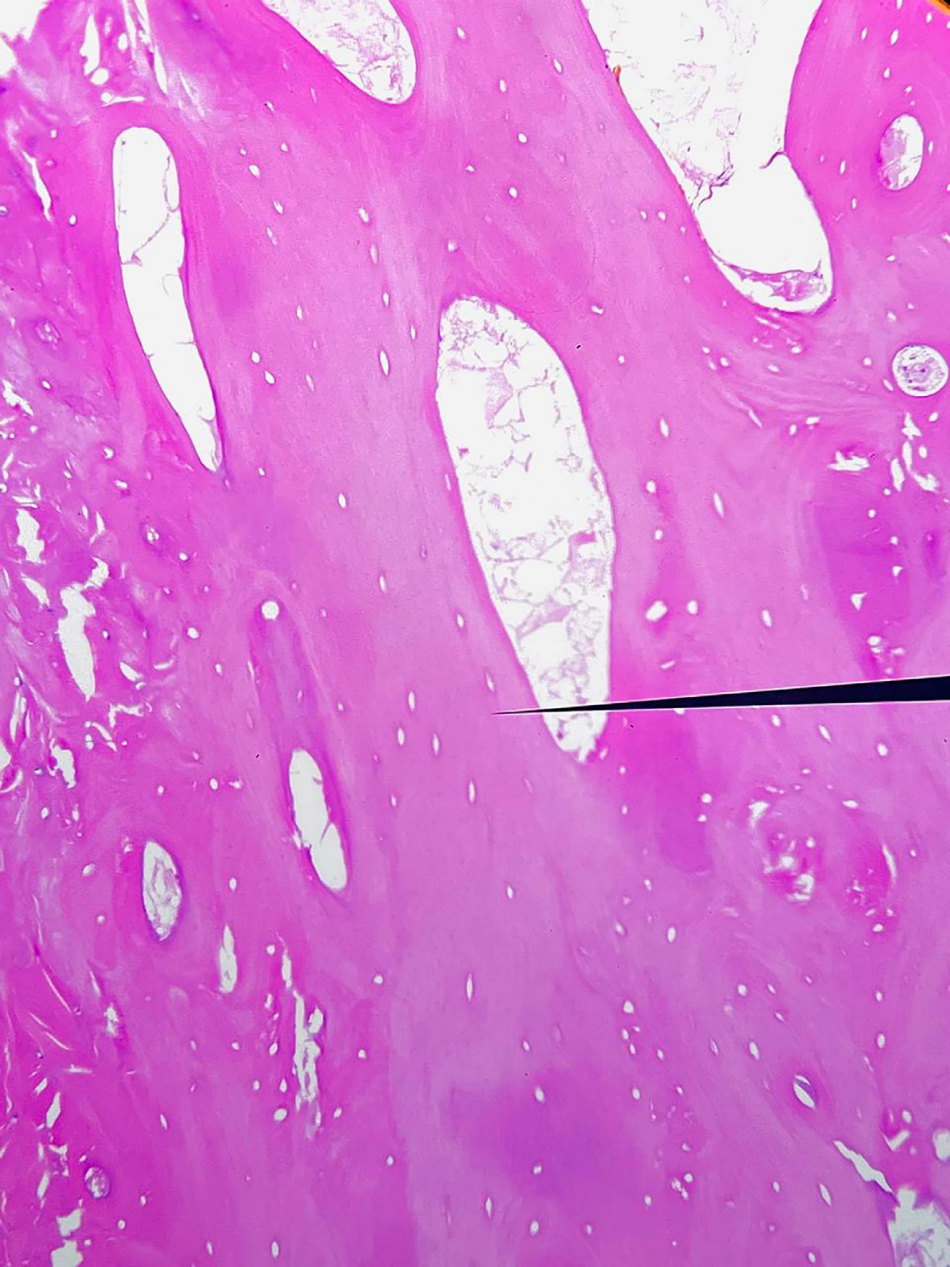
Histopathological section of the excised lateral hallucial sesamoid stained with hematoxylin and eosin (H&E), demonstrating devitalized bony trabeculae with absence of osteocytes in the lacunae and adjacent marrow space replacement by fibrous and fatty tissue. These findings are consistent with avascular necrosis.

MRI sequences revealed characteristic changes of osseous devitalization. Coronal and sagittal fat suppressed proton density (PD-FS) images showed hyperintensity of the lateral sesamoid, consistent with bone marrow edema secondary to AVN. The coronal T1-weighted image demonstrated corresponding hypointensity of the lateral sesamoid, suggestive of marrow edema. These findings together indicated chronic ischemic changes and bone necrosis, highlighting the value of MRI in early and definitive evaluation [[5], [6], [7]] (Fig. 1, Fig. 2, Fig. 3).

The treating team decided to perform a surgical excision of the necrotic left lateral sesamoid. The sesamoid bone was fragmented and lacked blood supply during the resection. A Chevron osteotomy was performed to correct the moderate hallux valgus deformity and redistribute the load to the intact medial sesamoid [7,8].

Histological assessment indicated the absence of osteocytes, the presence of marrow fibrosis, and reparative bone growth, hence corroborating the diagnosis of AVN. There was no evidence of osteomyelitis, tumor, or systemic illness.

The patient recovered without complications. At 3-month follow-up, she reported complete resolution of pain, improved toe alignment, and had resumed running at reduced intensity. There were no signs of hallux varus, transfer metatarsalgia, or functional limitations. The surgical site was well healed, and the osteotomy site showed good consolidation on follow-up imaging, reassuring of the successful surgical intervention.

## Discussion

Avascular necrosis (AVN) of the lateral hallucial sesamoid is an uncommon and often overlooked etiology of forefoot pain, especially among athletes. This case demonstrates a unique presentation of unilateral AVN in a patient with a history of contralateral surgical resection, allowing for an examination of the imaging, pathology, and biomechanical factors that influence diagnosis and treatment.

Initial plain radiographs of the left foot showed heterogeneous sclerosis and cortical irregularity of the lateral sesamoid, suggesting chronic osseous remodeling. The findings raised concern for AVN; however, plain films alone have insufficient sensitivity in the early stages of the disease [5]. MRI proved instrumental in confirming the diagnosis. Fat-suppressed proton density (PD-FS) images demonstrated increased signal intensity within the lateral hallucial sesamoid, consistent with bone marrow edema. This radiologic pattern correlates with histopathological findings of osteocyte loss, marrow fibrosis, and bone devitalization, providing a robust imaging-pathology match [5,6].

These MRI characteristics are hallmarks of sesamoid AVN, as they capture both the early inflammatory component and the later replacement of normal marrow with fibrous tissue. The use of MRI in this case allowed for precise localization and staging of the necrotic bone, thereby guiding timely surgical planning.

The imaging findings were closely aligned with the patient's clinical presentation: focal pain localized to the plantar aspect of the first metatarsophalangeal (MTP) joint, exacerbated by toe-off during gait, and unresponsive to conservative treatment. The patient's previous right lateral sesamoidectomy, evidenced by the absence of the bone on radiographs, supported the diagnostic suspicion of asynchronous bilateral disease.

During the operation, the left lateral sesamoid exhibited fragmentation and avascularity. Histopathological analysis confirmed AVN, revealing necrotic marrow, absence of osteocytes within the trabecular structure, and adjacent reparative fibrosis, all indicative of a chronic ischemic injury. No infectious, neoplastic, or inflammatory pathology was detected.

The choice to perform a Chevron osteotomy was influenced by the presence of a moderate hallux valgus deformity. The realignment of the first ray aimed to diminish abnormal shear forces at the medial sesamoid and first metatarsophalangeal joint [1,8]. Sesamoidectomy may alleviate pain; however, when combined with osteotomy in instances of joint malalignment, it may reduce future overload and maintain joint function. The strategy was considered successful following a complete symptom resolution and resumption of activity.

This case adds to the sparse literature on lateral sesamoid AVN, especially in individuals with a history of contralateral disease [5,9]. The etiology is multifactorial; however, the bilateral pattern in the absence of systemic risk factors indicates a potential biomechanical predisposition, such as altered plantar pressures or joint morphology. In this context, early MRI imaging and clinical suspicion are critical to prevent misdiagnosis or extended morbidity [1,3].

## Conclusion

Avascular necrosis of the hallucial sesamoids is a rare cause of forefoot pain often overlooked in athletes as the cause of metatarsalgia [1,3]. This case emphasizes the importance of using additional imaging methods for diagnosis, the significance of a thorough musculoskeletal examination, and the benefits of combining excision with realignment osteotomy for achieving optimal long-term function [8]. The occurrence of bilateral lateral sesamoid AVN makes this case unique and serves as an educational opportunity for the differential diagnosis of chronic foot pain in active patients.

## Patient consent

Written informed consent was obtained from the patient for publication of this case report and the accompanying images.
